# Real Remoldable Porcelain Including Moldable Gold, Silex, Carborundum, Corundum, Clays, Etc.

**Published:** 1907-12-15

**Authors:** Chas. H. Land


### REAL REMOLDABLE PORCELAIN INCLUDING MOLDABLE GOLD, SILEX, CARBORUNDUM, CORUNDUM, CLAYS, ETC.

BY CHAS. H. LAND, L. D. S.

Read before the Michigan State Dental Association, June 5th, 1907

The title of this paper is in contradistinction to several commercial products which usurp the name, having no adequate foundation in fact to support them. In this instance, I refer especially to certain compositions which are in reality formed of oxide of zinc and a small percent of vitrous material, combined with phosphoric acid, which, when scientifically nominated, are known as cements. In addition I will include certain compounds, silicates of soda, pottasium, etc. which are well established as glass, or semi-porcelains. Then we have the real pottery porcelain as distinct from china, stone ware, common pottery, pure clays, etc.

In order to obtain a true definition of vitrous masses, it will be necessary to consider them both and to have a practical knowledge of their chemistry and peculiarities when variously compounded. All vitrous masses, whether they are identified either as pottery, porcelain or glass, their natural origin is found in what is known as the feldspars, kaolin, and silex. For example, the formula of fine French china and porcelain may be kaolin 48 parts, feldspar 48 parts and chalk 4. In dental porcelains we leave out the chalk, increase the quantity of feldspar and silex, and reduce the kaolin.

I will now describe a series that I have arranged in groups. Combined they represent real moldable porcelains of glass, clay, sand, china, corundum or emery, carborundum, gold, silver, amalgam, and various other metals.

What I wish to convey, is that, all the above enumerated substances are put into a plastic form, so, that with your fingers alone they can be manipulated into an unlimited number of forms, we can also impress them into moulds of various kinds, and then when they are submitted to higher degrees of temperature they become hardened into durable and exceedingly valuable articles of utility, not only for dental purposes, but they cover a wonderful range of opportunities in various arts and manufactures.

I have made a series of specimens that I will undertake to describe in detail: Group A shows specimens of Brewsters body, shade 2 and 4; these show almost 40 per cent shrinkage, and 30 per cent less of color as compared with a high grade block body, both taken from the same mould.

Group B, Figure 14 of this group shows no loss of color, when in a state of partial vitrefaction. Here I must explain what I mean by partial vitrefaction or fusion: It is when a substance is so highly fused that it will flow into a homeogenous mass, like metals and their alloys, and will readily flow or mix thoroughly together. Silicate of soda or glass will do this when very highly heated, and then become transparent. On the other hand, if the same silicate of soda was only partially fused or vitrified, it would be either opaque or semi-translucent. If opaque, it might be recognized as a semi-porcelain, or an incomplete glass, and would not be entitled to the name of dental porcelain. The latter should consist of a compound mixture of particles, one of which is difficult of vitrefaction at very high temperatures, and the other particle that flow at a slightly lower temperature. The lower fusion mass merely acts as a binder; for example, take it in the case of carborundum, as shown in several specimens exhibited. In this instance, 25 per cent of block body is added to finely ground carborundum. These when mixed together and fired, form a mass hard enough to grind porcelain.

Figure 42 is common red or brick clay. Sand and clay keep very sharp outlines throughout the fire, and show very little shrinkage and is hard enough to grind glass if converted into a suitable wheel. The red clay when fired keeps very sharp outlines, and shrinks but little, but can be cut and carved with an ordinary pocket knife.

Group B represents a porcelain mould from which the casts of molar teeth were taken, one of them including a mould from the S. S. White block body, and three others are made from the high firing, consolidated tooth body. All these keep sharp outlines through the fire, take a most perfect glaze, hold their colors, and are the most translucent of any on the market today. Another illustration shows the same tooth composed of carborundum submitted to over 3000 degrees of temperature. This is substantially of the same combinations as our carborundum wheels, and is as hard. Notice that the shrinkage is so slight as to be imperceptible.

A number of moulded crowns are here shown which were moulded in plaster of Paris or brass moulds. They are high fired incisor tooth crowns, similar to the Davis, Whiteside, the Logan crown and common pivot teeth. In fact, with mouldable porcelain the day is fast approaching when each dentist from common plaster moulds can with the utmost facility turn out any form of manufactured teeth now on the market. I refer to the class that belong to the individual teeth, meaning not the rubber teeth or pin teeth for bridges and gold work, but the pivot class of teeth. I claim that we shall be able to do it better and cheaper, and not be inveigled into the scheme of paying $52.00 per pound for porcelain bodies that it does not cost $1.00 per pound to make, and which should sell at a fair profit at $2.00 per pound; nor will we have to pay 35 cents for a tooth with a hole in the end, when we can duplicate it dead easy for one cent, and turn out a tooth every five minutes ready to adjust to the mouth.

Another illustration represents a silver mould from which cast forms were taken made from ground high grade French porcelain or china ware. In one mould the French pottery porcelain shrinks about one-sixteenth, while the shrinkage of a cast made of the same pottery to which carborundum to the extent of one-half its bulk had been added, shows no perceptible shrinkage

These experiments are made to show that by the addition of gum chicle to porcelains of all grades they can be made mouldable at pleasure, or can be cast into definite forms and baked to vitrification without change of form or loss of detail, providing a true or high fusing porcelain be used. The higher the fusing point the sharper the detail, and better the form and the less shrinkage. The addition of infusible materials like corundum, emery and corborundum seem to materially lessen the shrinkage.

### DISCUSSION.

Dr. J. M. Thompson. Through the courtesy of Dr. Land, I have been enabled to witness some of these moldable demonstrations. I have taken pains to do a little of it myself. Two or three times I have demonstrated to some of my friends that have been in my office from time to time what could be done along these lines with this material.

I cannot give you the information from my own experience as Dr. Land can. In places where we have to make porcelain supports, or are called upon to assemble parts, this material can be used to advantage. I think as we become more familiar with the material we shall find it very helpful. The Doctor says we can use it in almost any place we want to. We can take an impression of the mouth, or several teeth in the mouth; if we want two or three teeth joined together we can do it. I am very sorry I have not had more time to experiment with it before coming to the meeting.

I wish that we all could have some experience with Dr. Land's work in this material, as I have had the last two or three weeks, and we should then realize its value.

Dr. Worboys. There is one question I should like to ask, and it is this: Supposing we have a root prepared and are going to put a crown on it; we have a post fitted into the stump, and after forming the moldable porcelain to that surface and baking it, will it shrink down or will it have to be remodeled?

Dr. Land. You must take the moldable silicia and paint it over the surface of the porcelain.

Dr. Roach, of Chicago. I wish to assure you that it has afforded me great pleasure to hear this paper and to have seen this magnificent floral work at the hands of Dr. Land. I have known Dr. Land for a great many years, known of him in his work a good many years, and have always looked to Dr. Land as one of our leaders in porcelain work. He has been an inspiration to me in my feeble efforts along the line of porcelain work. This is along the line of work that I have been experimenting for a number of years, and is in the same direction that others are working. Dr. Land has eliminated some of the objections that have been made to the use of porcelain; they have been objections that most of us have been unable to overcome.

I think Dr. Land has made, perhaps a little unconsciously in the title of his paper a direct slap at me in the name of Moldable Porcelain. I don't think it makes much difference with the majority of us as to the name. I think I shall refrain from any discussion of the matter, but I hope to be able to convince Dr. Land that my moldable porcelain is a true name; that the materials are such as shall justify the name.

I have no doubt but that the material he has given us is a very valuable one. I am always willing to take off my hat to the other fellow if he gives us something better. I am of the belief that Dr. Land has given us an idea that will be very valuable in our practice.

